# Membrane-based microfluidic solvent extraction of Ga-68 from aqueous Zn solutions: towards an automated cyclotron production loop

**DOI:** 10.1186/s41181-023-00195-2

**Published:** 2023-05-05

**Authors:** Svenja Trapp, Tom Lammers, Gokce Engudar, Cornelia Hoehr, Antonia G. Denkova, Elisabeth Paulssen, Robin M. de Kruijff

**Affiliations:** 1 Department of Radiation Science and Technology, Reactor Institute Delft, Delft University of Technology, Mekelweg 15, 2629 JB Delft, The Netherlands; 2 Life Sciences Division, TRIUMF, Vancouver, BC Canada; 3 Department of Chemistry and Biotechnology, Aachen University of Applied Science, Juelich, Germany

**Keywords:** Microfluidic solvent extraction, Ga-68, Cyclotron production, Medical radionuclide production, Zinc nitrate liquid target, Metal contaminants

## Abstract

**Background:**

The radionuclide Ga-68 is commonly used in nuclear medicine, specifically in positron emission tomography (PET). Recently, the interest in producing Ga-68 by cyclotron irradiation of [^68^Zn]Zn nitrate liquid targets is increasing. However, current purification methods of Ga-68 from the target solution consist of multi-step procedures, thus, leading to a significant loss of activity through natural decay. Additionally, several processing steps are needed to recycle the costly, enriched target material.

**Results:**

To eventually allow switching from batch to continuous production, conventional batch extraction and membrane-based microfluidic extraction were compared. In both approaches, Ga-68 was extracted using N-benzoyl-N-phenylhydroxylamine in chloroform as the organic extracting phase. Extraction efficiencies of up to 99.5% ± 0.6% were achieved within 10 min, using the batch approach. Back-extraction of Ga-68 into 2 M HCl was accomplished within 1 min with efficiencies of up to 94.5% ± 0.6%. Membrane-based microfluidic extraction achieved 99.2% ± 0.3% extraction efficiency and 95.8% ± 0.8% back-extraction efficiency into 6 M HCl. When executed on a solution irradiated with a 13 MeV cyclotron at TRIUMF, Canada, comparable efficiencies of 97.0% ± 0.4% were achieved. Zn contamination in the back-extracted Ga-68 solution was found to be below 3 ppm.

**Conclusions:**

Microfluidic solvent extraction is a promising method in the production of Ga-68 achieving high efficiencies in a short amount of time, potentially allowing for direct target recycling.

**Graphical Abstract:**

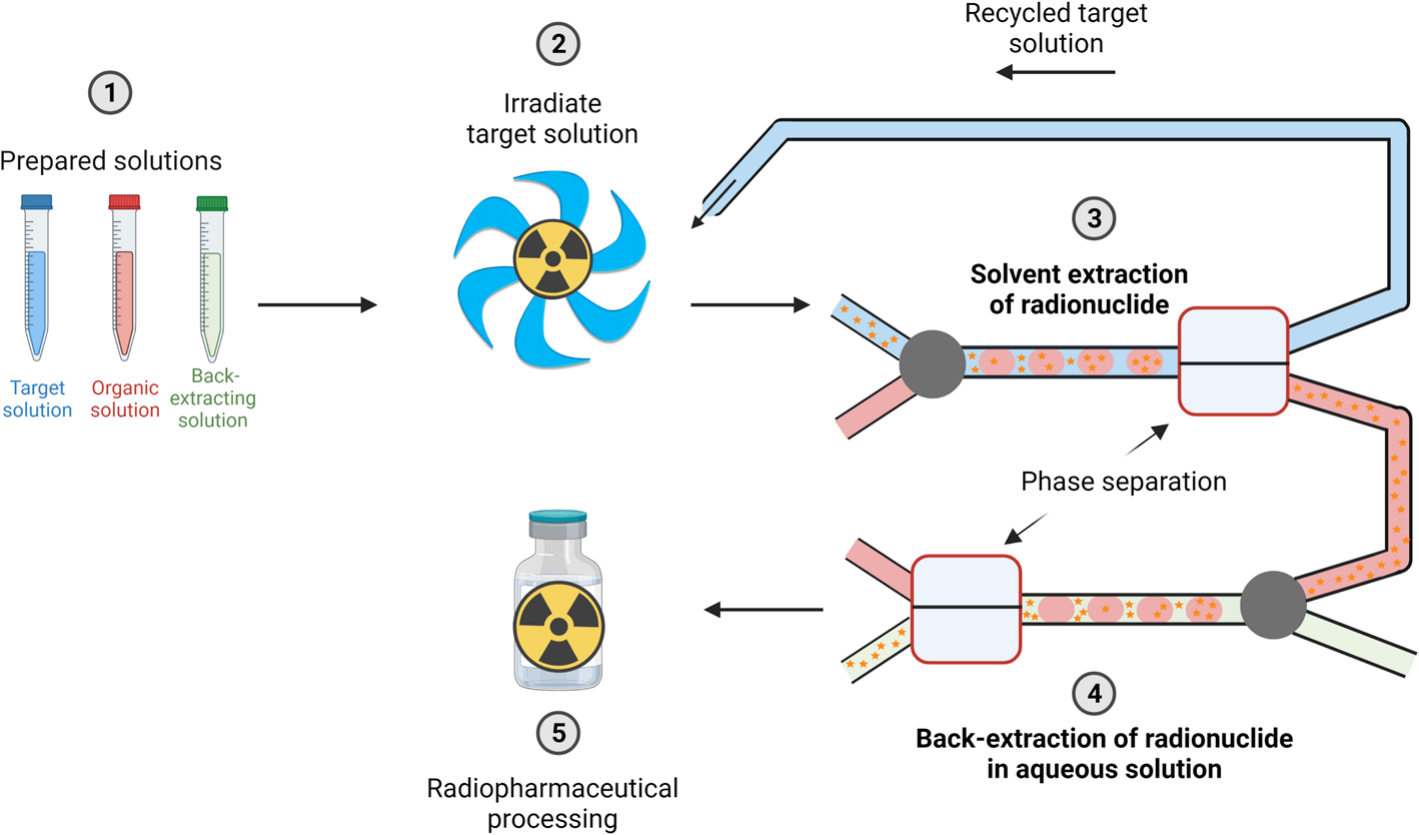

## Background

The use of ^68^Ga-labelled radiopharmaceuticals has become increasingly popular in nuclear medicine over the last 2 decades. Ga-68 has ideal decay characteristics for high quality PET imaging, such as a positron yield of 89.1% and an average β^+^ energy of 836 keV (Sanchez-Crespo 2013) while the short half-life of 67.71 min minimizes the total dose to the patient and medical personnel. Since its radiolabelling chemistry is well understood, it can easily be applied in a variety of radiopharmaceuticals (Banerjee and Pomper 2013; Maecke and André 2007; Isabel and Prata 2012). Recently, a new Ga-68 prostate-specific membrane antigen ligand ([^68^Ga]Ga-PSMA-11) for PET imaging of metastasized prostate cancer was approved by US Food & Drug Administration (2022), which can detect significantly more prostate lesions than the previously used [^18^F]fluciclovine (Calais et al. 2019). Furthermore, ^68^Ga-labelled fibroblasts activation protein inhibitors (FAPI) showed remarkable results in PET imaging of up to 30 different tumor types (Kratochwil et al. 2019). Currently, over 500 clinical trials are using ^68^Ga-labelled compounds as a diagnostic agent (Trials 2022) showing that the applications of ^68^Ga in nuclear medicine are steadily expanding. An increase in demand in Ga-68 can therefore be expected. To date, Ga-68 is mostly produced by Ge-68/Ga-68 generators, due to their easy handling and accessibility for hospitals without an irradiation infrastructure (Maecke and André 2007; Rösch 2013). But several limitations of these generators, such as the high costs, an increasing shortage in the target material to produce Ge-68, the limited amounts of Ga-68 that can be eluted from a generator in a day (Rösch 2013; Lambrecht 1983; Dash and Chakravarty 2019), as well as the high amounts of radioactive Ge-68 waste after the shelf-life of a generator is reached, result in the necessity to investigate other Ga-68 production routes. Proton irradiation of Zn-68 could significantly amplify the production of Ga-68 by the ^68^Zn(p,n)^68^Ga reaction (Velikyan 2015). Several studies on the cyclotron production of Ga-68 from solid and liquid Zn targets exist to date (Pandey et al. 2014a, 2019; Riga et al. 2018; Alves et al. 2017a; Rodnick et al. 2020; Engle et al. 2012). Although the irradiation of liquid targets results in lower production yields compared to solid targets, they benefit from reduced target preparation steps pre and post irradiation, while still producing higher activities than generators. Most commercial Ge-68/Ga-68 generators produce a maximum of 1.85 GBq per elution, although this has recently increased with the development of a Ge-68/Ga-68 generator producing up to 3.7 GBq per elution (Radiopharma. 2021). These generators can only be eluted between one to three times per working day, dependent on the generator, loaded Ge-68 radioactivity, and the generators age (Velikyan 2015). In comparison, Alves et al. (2017b) reported a yield of 6 GBq Ga-68 in a single 45 min irradiation of a [^68^Zn]Zn liquid target with a 12.9 MeV medical cyclotron and a beam current of 45 µA, which can be upscaled to produce up to 40 GBq per irradiation by increasing the concentration of [^68^Zn]Zn in the liquid target. Therefore, a single irradiation of a [^68^Zn]Zn liquid target could produce a more than 10-times higher yield than the best currently available generators, making it a worthy alternative for hospitals that have a medical cyclotron (up to 12 MeV (Siikanen et al. 2021)) infrastructure available. But at the same time, current extraction methods of Ga-68 from the target solution after irradiation are multistep procedures that can lead to a significant loss of activity due to natural decay and do not enable direct recycling of the costly, enriched target material. Current literature on Ga-68 production using liquid targets focus mostly on the purification of Ga-68 using a combination of anion and cation exchange resins. As a first step cation exchange resins are used (e.g., hydroxamate, DOWEX 50W-X8, AG-50W-X8) to trap Ga-68 and wash most [^68^Zn]Zn. Enriched [^68^Zn]Zn must be recovered and processed from the washing solutions before it can be reused for irradiation. Next, Ga-68 is eluted from the first column in large volumes of highly concentrated HCl solutions and loaded onto an anion exchange resin (e.g., Biorad 1X8, AG-1X-8, DGA, TK200) from which Ga-68 can be eluted in 0.1 M HCl or water, depending on the resin (Pandey et al. 2014a, 2019; Riga et al. 2018; Alves et al. 2017a, b). These methods usually lead to a purified [^68^Ga]GaCl_3_ solution in 30–60 min with yields between 78 and 90%. Microfluidic solvent extraction presents a very attractive, fast alternative to column chromatography (Martini et al. 2019) for the separation of Ga-68 from the target solution, with an efficient two-step procedure that potentially enables extraction automatization and direct target recycling. In this approach, the target solution is not changed after irradiation (e.g. change of acid concentration), and can therefore potentially be directly recycled without any further processing steps as shown in Graphical Abstract. While several studies exist on microfluidic solvent extraction on other medical radionuclides (Trapp et al. 2023; Martini et al. 2021; Dalmázio and Oehlke 2017), Pedersen et al. (2019) were the first to develop a microfluidic solvent extraction method to separate Ga-68 from a ZnCl_2_ in HCl target solution. However, the rate of radiolysis of water in hydrochloric acid-based target solutions is known to lead to rapid pressure increase upon cyclotron irradiation, potentially forcing the abortion of irradiations (Pandey et al. 2014a). It can also lead to strong corrosion of the Havar^©^ foil used in the liquid target body (Oehlke et al. 2015). Hence, irradiation of zinc liquid targets focusses almost exclusively on zinc nitrate solutions in dilute nitric acid target (do Carmo et al. 2020; Pandey et al. 2019; Riga et al. 2018; 2017a, b; Oehlke et al. 2015) due to the ability of nitrates to scavenge free radicals produced during the irradiation of water, thereby reducing the target pressure (Pandey et al. 2014b). Zhuravlev et al. (2022) took the first steps in the development of a membrane-based microfluidic solvent extraction of Ga-68 from zinc nitrate solutions using an arylamino phosphonate leading to an extraction efficiency of 80% in flow. However, this compound is not commercially available and was newly synthesized for their approach. In our study, we present a highly efficient microfluidic solvent extraction method for the selective extraction of Ga-68 from zinc nitrate liquid target solutions using a commercially available chelator, therefore avoiding the need for complex synthesis. Batch experiments are conducted to determine equilibrium extraction efficiencies for different concentrations of potential target solutions, followed by microfluidic experiments including a proof-of-principle run with a irradiated target solution.

## Materials and methods

### Materials and analytical methods

N-benzoyl-N-phenylhydroxylamine (BPHA; reagent grade < 98%; CAS: 304-88-1) and zinc nitrate hexahydrate (Zn(NO_3_)_2_·6H_2_O; reagent grade < 98%) were purchased from ACROS ORGANICS (VWR, Amsterdam, the Netherlands), nitric acid (HNO_3_; Reag. ISO, Reag. Ph. Eur), hydrochloric acid (HCl; technical grade) and chloroform (Reag. Ph.Eur., ACS; stabilized with 0.6% ethanol) and were purchased from Sigma Aldrich (Merck Sigma, Zwijndrecht, the Netherlands). Acid dilution were performed in ultrapure water, obtained from a Milli-Q Advantage system. An Eckert & Ziegler IGG100 GMP Ge-68/Ga-68 generator was generously supplied by Erasmus MC, the Netherlands. Zn-69m (t_1/2_ = 13.7 h, gamma energy = 438 keV (95%)) was produced by neutron irradiation of 6 mg [^nat^Zn]Zn foil at the Hoge Onderwijs Reactor (HOR) of the Reactor Institute Delft (the Netherlands). After subsequent cooling for 10 h, the foil was dissolved in 0.1 mL 8 M HNO_3_ and diluted to 10 mL with distilled water. Microfluidic extractions were executed with a SEP-10 membrane separator with hydrophobic membranes (pore size: 0.45 µm) from Zaiput Flow Technologies (Waltham, Massachusetts, USA). AL-1000 Programmable Syringe pumps (941-371-1003) were purchased from World Precision Instruments Inc. and used to deliver solutions to the membrane separator via PTFE tubing (100 cm length, 0.03 inch inner diameter). A slug flow was achieved by using a Microfluidic Y Connector PEEK from IDEX Health & Science (Oak Harbor, Washington, USA). The Vortex-Genie 2 used for batch extractions was purchased from Scientific Industries, Inc (Bohemia, New York, USA). Metal contaminations were measured with an ICP-OES Optima 8000 from Perkin Elmer (Groningen, The Netherlands). The Wallac Wizard^2^ 3″ 2480 Automatic Gamma Counter from Perkin Elmer (Groningen, The Netherlands) was used for gamma-radiation measurements.

### Experimental methods

#### Batch extraction

To optimize the Ga-68 extraction, aqueous solution concentrations ranging from 1 to 5 M [^nat^Zn]Zn(NO_3_)_2_ in 0.01 M–1 M HNO_3_ were tested, according to reported target concentrations in the literature (do Carmo et al. 2020; Pandey et al. 2019; 2017a, b). 10–15 kBq of Ga-68 from a Ge-68/Ga-68 generator was added to each aqueous solution to trace Ga-68 extraction and 3–5 kBq Zn-69m were added to trace Zn-coextraction. BPHA was chosen as the extractant due to its capability to extract Ga^3+^ from highly acidic media and known kinetics of the BPHA-Ga complex (Riedel 1973; Lyle and Shendrikar 1965; Morroni et al. 2004). The organic solution used consisted of 0.2 M BPHA in chloroform. Additionally, dithizone was selected as an extractant to selectively extract Cu contaminations from the target solution. Conventional batch extraction experiments were executed in Eppendorf vials with a 1:1 volumetric ratio of aqueous and organic phase with volumes of 0.5 mL each. The Eppendorf vials were shaken with the Vortex for 10 min or 1 min with BPHA or Dithizone, respectively, to ensure extraction equilibrium was reached. Afterwards, the organic phase was separated by pipetting. The Ga-68 and Zn-69 m radioactivity in the aqueous solution before extraction (A_initial_) sand organic phase after extraction (A_organic_) was measured with the Wallac Wizard^2^ 3″ 2480 Automatic Gamma Counter and corrected for decay. The extraction efficiencies (EE%) were calculated according to:

1 $${\text{EE\% }} = { }\frac{{{\text{A}}_{{{\text{organic}}}} }}{{{\text{A}}_{{{\text{initial}}}} }}\cdot100\%$$

To evaluate back-extraction of Ga-68 from the organic phase into an aqueous phase, different concentrations of 0.1 M to 6 M HCl were added to the organic phase, again with a 1:1 volumetric ratio. The Ga-68 activity of the HCl solutions was measured separately after back-extraction (A_HCl_), corrected for decay, and back-extraction efficiencies (BEE%) were calculated according to:

2 $${\text{BEE\% }} = { }\frac{{{\text{A}}_{{{\text{HCl}}}} }}{{{\text{A}}_{{{\text{organic}}}} }}\cdot100\%$$

Each extraction experiment was executed in triplicate and errors are given as one standard deviation of the mean. Since Fe, Cu, Ni, Co and Mn are often found as non-isotopic impurities after cyclotron irradiation of [^68^Zn]Zn(NO_3_)_2_ solutions (Riga et al. 2018), their co-extraction in the developed extraction system was investigated following the same procedure. Therefore, 0.1 mM stable Fe(III), Cu, Ni, Co and Mn were added to the zinc solution. Their co-extraction was measured using ICP-OES.

#### Microfluidic solvent extraction

**Fig. 1:**
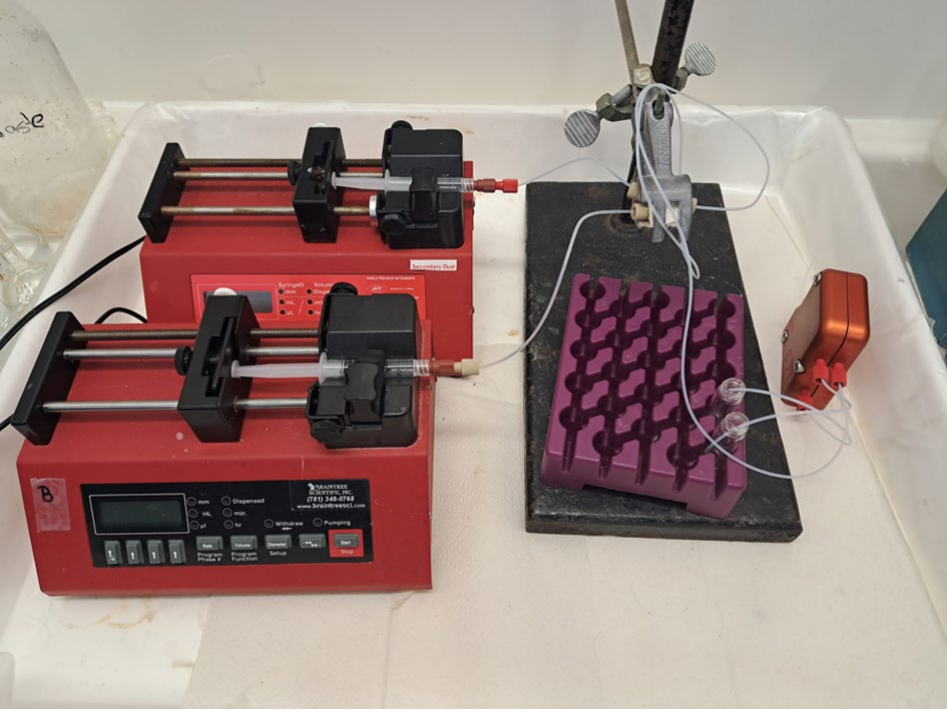
Microfluidic solvent extraction set-up using a Zaiput Sep-10 membrane separator. The slug flow is created by the fluidic y-connector

To achieve complete phase separation after the extraction, a membrane-based separation device (Zaiput Membrane Separator (Zaiput Flow technologies 2022)) was used. Solvent extraction was executed in microfluidic PTFE tubing of 100 cm length and an inner diameter of 0.03 inch. The tubing was connected to two syringes, containing the aqueous and organic solutions (Fig. 1). A constant flow of the aqueous and organic solutions was achieved using syringe pumps and a slug flow was created with a microfluidic Y-connector, to increase the surface-to-volume ratio of the aqueous and organic solution, maximizing extraction. All microfluidic solvent extraction experiments were performed at a 1:1 volumetric ratio with a flow rate of 40 μL/min. The radioactivity of Ga-68 was again measured with the Wallac Wizard^2^ 3″ 2480 Automatic Gamma Counter in the aqueous (A_aqueous_) and organic solution (A_organic_) after the extraction. Extraction efficiencies (EE%) were calculated according to:

3 $${\text{EE\% }} = { }\frac{{{\text{A}}_{{{\text{organic}}}} }}{{{\text{A}}_{{{\text{organic}}}} + {\text{A}}_{{{\text{aqueous}}}} }}\cdot100\%$$

Additionally, back-extraction was investigated in the microfluidic set-up with 2 M and 6 M HCl and varying flow rates from 50 to 150 μL/min. Back-extraction efficiency (BEE%) was calculated according to:

4 $${\text{BEE\% }} = { }\frac{{{\text{A}}_{{{\text{HCl}}}} }}{{{\text{A}}_{{{\text{organic}}}} + {\text{A}}_{{{\text{HCl}}}} }}\cdot100{\text{\% }}$$

with A_HCl_ = Ga-68 activity in the aqueous HCl solution after back-extraction and A_organic_ = Ga-68 activity in the organic solution after back-extraction. Solutions used for back-extraction were collected to determine Zn, Cu, Ni, Co, Fe and Mn contamination by ICP-OES. All experiments were executed in triplicate and errors are given as one standard deviation of the mean. All measured activities were corrected for decay.

#### Cyclotron targetry and irradiation

Three irradiations on 2 M [^nat^Zn]Zn(NO_3_)_2_ solutions in 0.01 M HNO_3_ were executed on TRIUMF’s TR13 cyclotron, a 13 MeV self-shielded, negative hydrogen ion cyclotron. This target concentration was selected due to its common use in literature (Pandey et al. 2014a, 2019; Riga et al. 2018; Alves et al. 2017a; Rodnick et al. 2020) as well as extraction performance. The target solutions were irradiated in a siphon-style niobium body target with an internal expansion chamber as described by Hoehr et al. (2012) and Lowis et al. (2021). The target chamber has a volume of 1.48 mL and is separated from the cyclotron vacuum by a double foil window with a water-cooling jacket on the back and a helium jet cooling on the front site of the target. These two foils (25 μm thick aluminium outside of target and 38 μm HAVAR® foil inside of target) cause the proton beam to get degraded to 12 MeV. Before the start of the irradiation the internal expansion chamber was pressurized to 200 psi. A 10 μA proton beam was applied for 30 min (n = 3). After irradiation and unloading, 5 mL of radioactive solution was obtained. 2.5 mL was loaded into a syringe and microfluidic extractions were executed as described above with a flow rate of 40 µL/min. The extraction efficiency of ^68^Ga was calculated according to Eq. 3 using the characteristic peak at 1077.34 keV.

## Results

### Batch extraction

**Fig. 2:**
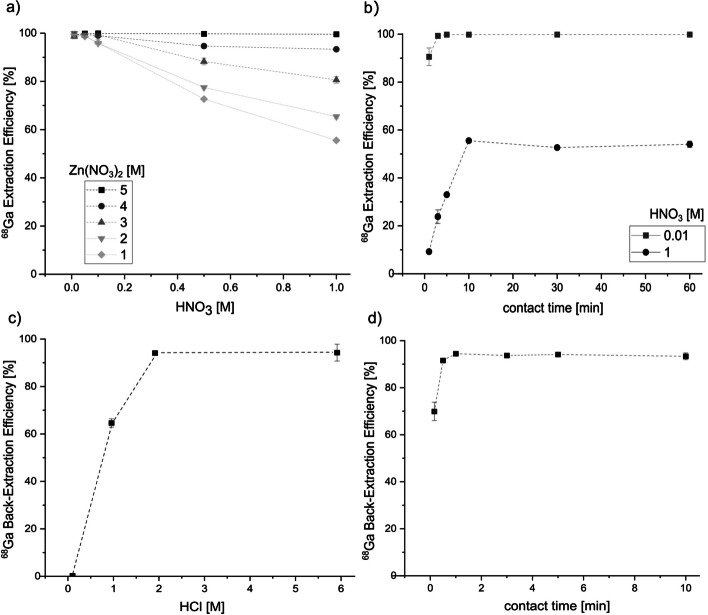
**a** Extraction efficiencies for batch extraction of Ga-68 from various Zn(NO_3_)_2_ solutions in HNO_3_ for a contact time of 10 min. **b** Extraction efficiencies over time for batch extraction of Ga-68 from 1 M Zn(NO_3_)_2_ in 0.01 and 1 M HNO_3_ solutions. **c** Back-extraction efficiencies for the batch extraction of Ga-68 into different HCl concentrations within 1 min contact time. **d** Back-extraction efficiencies obtained for different contact times for the back-extraction of Ga-68 into 2 M HCl. Error bars represent one standard deviation of the mean

Batch extraction of different concentrations of target solutions, ranging from 1 to 5 M Zn(NO_3_)_2_ in 0.01–1 M HNO_3_, were performed to investigate optimal target concentrations to optimize Ga-68 extraction efficiencies. 5 M Zn(NO_3_)_2_ solutions all showed maximum extraction efficiencies of over 99.3%. However, a trend of decreasing extraction efficiencies can be observed with increasing HNO_3_ concentrations for lower Zn(NO_3_)_2_ concentrations (Fig. 2a). Extraction efficiencies in the solutions containing 1 M Zn(NO_3_)_2_ range from 99.5% ± 1.2% to 55.5% ± 0.6% in 0.01 M and 1 M HNO_3,_ respectively. Increasing the contact time to up to 60 min did not increase extraction efficiencies (Fig. 2b), indicating that the results in Graphical Abstract represent equilibrium. Therefore, lower HNO_3_ concentrations of 0.01 M HNO_3_ are required to achieve high extraction efficiencies, exceeding 99% for all studied Zn(NO_3_)_2_ concentrations. Zinc co-extraction was measured to be 0.09% ± 0.06% from a 2 M Zn(NO_3_)_2_ solution in 0.01 M HNO_3_. Back-extraction efficiencies of 94.5% ± 0.6% could be achieved within 1 min by using 2 M HCl as back-extracting agent. Higher HCl concentrations showed to achieve similar results, while lower HCl concentrations led to decreasing back-extraction efficiencies (Fig. 2c).

Increasing the contact time to more than 1 min did not show increased back-extraction efficiencies (Fig. 2d) indicating equilibrium was reached within such a short time.

#### Co-extraction of other metal impurities

Co-extraction of non-isotopic metal impurities from a 2 M ^nat^Zn(NO_3_)_2_ solution in 0.01 M HNO_3_ into BPHA and dithizone was investigated in batch (Table 1). Cu and Fe are co-extracted by BPHA with 99.3 ± 0.4% and 99.45 ± 0.03% efficiency respectively, using a contact time of 5 min. Mn, Co and Ni are not extracted in significant amounts by BPHA (< 5%). Using 10 mM dithizone in chloroform, Cu impurities could be extracted from the target solution within 1 min of vortexing time with an efficiency of 99.91 ± 0.06% without co-extracting Ga-68, giving the opportunity to extract Cu-61, which is simultaneously produced by proton irradiation of ^nat^Zn (Council of Europe 2019; International Atomic Energy Agency 2018). All other tested impurities showed very little to no extraction using dithizone (< 10%).

**Table 1:** Extraction efficiencies for different metals using BPHA (5 min contact time) and dithizone (1 min contact time) from 2 M Zn(NO_3_)_2_ in 0.01 M HNO_3_. Fe(III), Cu, Mn, Co and Ni ions were added to the Zn(NO_3_)_2_ solution at concentrations of 0.1 mM. The reported uncertainty is given as one standard deviation of the mean (n = 3)

	Zn [%]	Ga [%]	Fe [%]	Cu [%]	Mn [%]	Co [%]	Ni [%]
BPHA	0.09 ± 0.06	99.6 ± 0.3	99.45 ± 0.03	99.3 ± 0.4	0.8 ± 0.3	4.1 ± 1.6	1.9 ± 1.2
Dithizone	0.31 ± 0.19	0.03 ± 0.02	9.3 ± 3.0	99.91 ± 0.06	3.3 ± 2.3	5.1 ± 1.5	7.3 ± 1.9

#### Metal contamination in final solutions

**Fig. 3:**
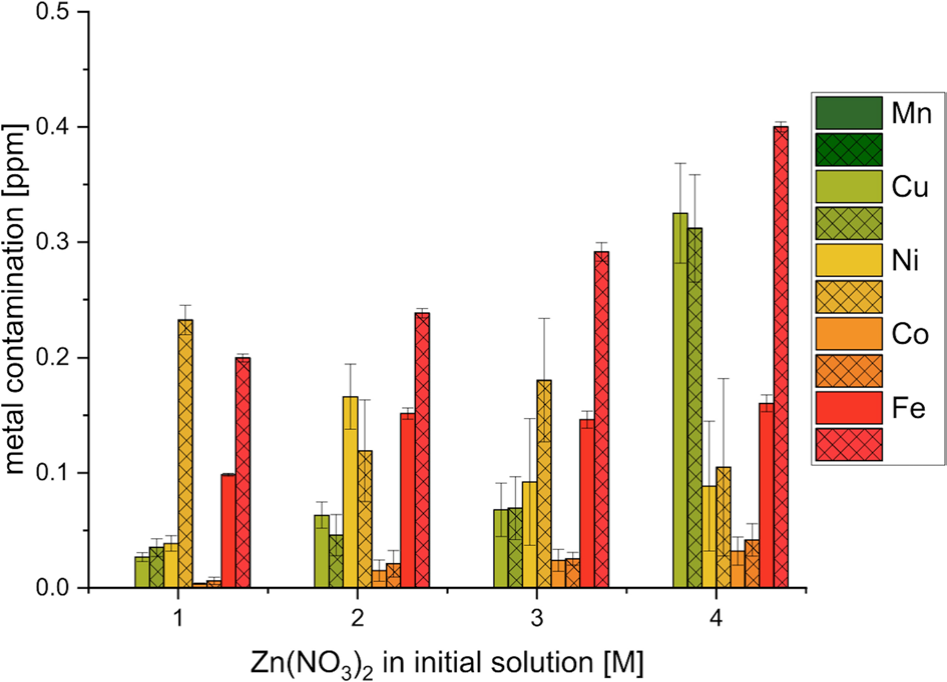
Mn, Cu, Ni, Co and Fe contaminations in back-extracted solutions (plain = into 2 M HCl; crossed = into 6 M HCl) after extraction from 2 M Zn(NO_3_)_2_ solution in 0.01 M HNO_3_. Mn was below the detection limit of 10 ppb. Error bars represent one standard deviation of the mean (n = 3)

Metal contamination in the back-extracted 2 M and 6 M HCl solutions after batch extraction from 2 M [^nat^Zn]Zn(NO_3_)_2_ in 0.01 M HNO_3_ was measured by ICP-OES (Fig. 3). All measured metal contaminations, including Cu, Ni, Co, Fe, and Mn were below 0.5 ppm, with Mn being below the detection limit (< 10 ppb).

### Microfluidic extraction

**Fig. 4:**
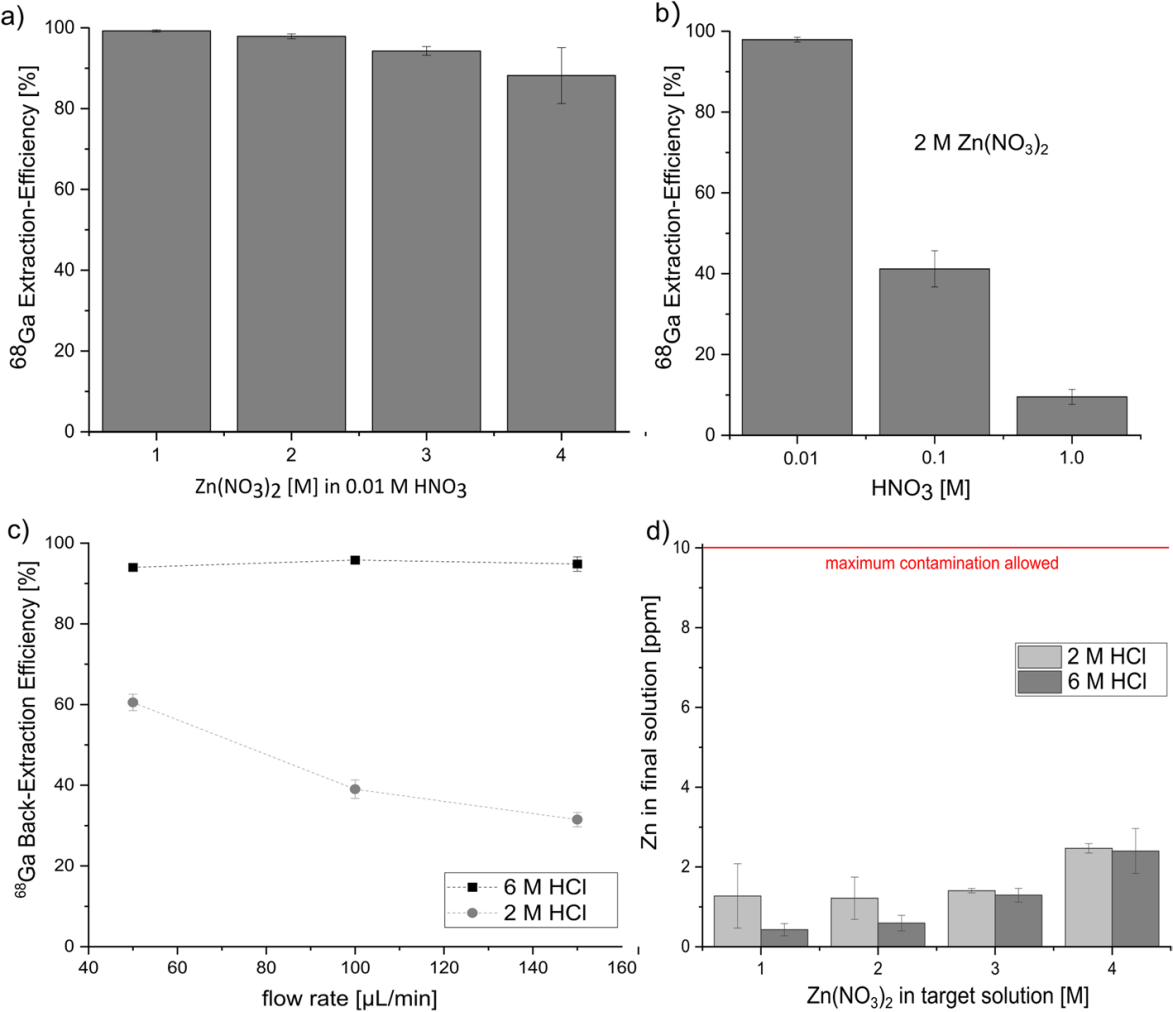
**a** Extraction efficiencies for microfluidic extraction of Ga-68 from various Zn(NO_3_)_2_ solutions in 0.01 M HNO_3_. The flow rate was 40 µL/min for both solutions. **b** Extraction efficiencies for microfluidic extraction of Ga-68 from 2 M Zn(NO_3_)_2_ solutions with varying nitric acid concentrations. The flow rate was 40 µL/min. **c** Back-extraction efficiencies for microfluidic back-extraction of Ga-68 into 2 M and 6 M HCl with varying flow rates. **d** Zn contamination in final HCl solutions after back-extraction. Error bars represent one standard deviation of the mean (n = 3)

Microfluidic extraction experiments were performed with varying Zn(NO_3_)_2_ and nitric acid concentrations (Fig. 4). The results show a decreasing extraction efficiency with increasing Zn(NO_3_)_2_ concentrations (Fig. 4a). While 1 M Zn(NO_3_)_2_ resulted in an extraction efficiency of 99.2% ± 0.3%, the 4 M Zn(NO_3_)_2_ solution only showed an efficiency of 88.2% ± 6.9% when the same flow rate of 40 µL/min was applied. Increasing the HNO_3_ concentration significantly impacted the extraction efficiency (Fig. 4b), decreasing it, for instance, for 2 M Zn(NO_3_)_2_ from 97.9% ± 0.6% in 0.01 M HNO_3_ to 41.2% ± 4.5% and 9.5% ± 1.8% in 0.1 M and 1 M HNO_3_, respectively. Very different back-extraction efficiencies were found using either 2 M or 6 M HCl as back-extraction solutions. While 6 M HCl consistently showed efficiencies exceeding 94% for varying flow rates between 50 and 150 μL/min, 2 M HCl achieved only 60.5% ± 2.0% decreasing to 31.5% ± 1.8%, for 50 and 150 μL/min, respectively (Fig. 4c). Zn contaminations did not exceed 3 ppm for all tested target solutions concentrations (Fig. 4d), conforming to IAEA TecDoc standards (International Atomic Energy Agency 2018). To estimate the contamination according to the European Pharmacopoeia (Council of Europe 2019), where a maximum of 10 μg/GBq is allowed, we compared our results to a production of Ga-68 from enriched [^68^Zn]Zn by Alves et al. (2017a). They irradiated a 2.2 M Zn(NO_3_)_2_ solution, which resulted in an Ga-68 activity of 25 GBq at EOB. Taking 2 h of decay into consideration for processing of the target solution and production of radiopharmaceuticals, while considering a Zn contamination of 3 ppm after the presented separation methods, the resulting contamination would equal to 2.5 μg/GBq. While this is just a approximation and results highly depend on irradiation parameters and target concentrations (Pandey et al. 2019), the results look promising to fulfil the European Pharmacopoeia standards (Council of Europe 2019).

Additionally, a microfluidic extraction was performed on a cyclotron irradiated 2 M Zn(NO_3_)_2_ solution in 0.01 M HNO_3_. Extraction efficiencies of 97.0% ± 0.4% (n = 3) were obtained and are within uncertainty identical to the results observed with low activity solutions of 97.9% ± 0.6%. However, it was observed that the membranes used in these experiments were sensitive to radiolysis and started to break down after being in contact with a target solution containing between 500 and 600 MBq of Ga-68 for more than 20 min.

## Discussion

### Extraction behaviour

During cyclotron irradiation of liquid targets high amounts of gases can be produced, leading to a drastic pressure increase inside the target body. Adding nitric acid to Zn target solutions can reduce the pressure build up by scavenging free radicals. Several different concentrations of nitric acid in target solutions have been used successfully in the literature (Pandey et al. 2019). Therefore, in this study multiple nitric acid concentrations (0.01–1 M) in the target solutions were studied. These target solutions have a pH between − 1.3 and 1.7, which is generally not preferred for solvent extraction purposes. Most conventional extractants only efficiently extract ions at higher pH (> pH 2) (Vadasdi 1969; Dhond and Khopkar 1976; Bhattacharya et al. 2007; Inoue et al. 1988; Lee et al. 2002; De and Asit 1967) or from highly acidic chloride solutions that will lead to the formation of chloride species (Ahmed et al. 2013; Song et al. 2020; Katsuta et al. 2012; Maljkovic et al. 1990). However, in the solutions tested in this study, the dominant species is Ga^3+^ with minor amounts of Ga(OH)^2+^ (calculated with *CHEAQS Next* software (Verweij Wi. CHEAQS Next (0.2.1.8))). Therefore, most commercial extractants are not applicable. BPHA on the other hand has the ability to bind to Ga^3+^ and Ga(OH)^2+^ according to reactions in Eqs. 5 and 6 (adapted from Morroni et al. (2004) with L representing the ligand BPHA and M the metal Ga), making it a suitable extractant for extracting ^68^Ga from Zn(NO_3_)_2_ liquid targets.

5 $${\text{HL}} + {\text{M}}^{3 + } \to {\text{MHL}}^{3 + }$$

6 $${\text{HL}} + {\text{M(OH)}}^{2 + } \to {\text{ML}}^{2 + } + {\text{H}}_{2} {\text{O}}$$

In the presented experiments, a clear effect of the amount of H^+^ ions in the solution can be observed, indicating that lower acid concentration in the target solution (0.01–0.1 M HNO_3_) are generally preferable to achieve the highest equilibrium extraction efficiency in the shortest contact time. Intramolecular hydrogen bonding was proposed as a reason for this reduction in extraction efficiencies (Morroni et al. 2004). However, increasing the Zn(NO_3_)_2_ concentrations in the target solutions leads to an increase in Ga-68 extraction efficiency even when higher acid concentrations are used, probably due to the ‘salting-out effect’. This effect describes the positive effect of salt concentration on the partition coefficient and the separation factor of the extractant during the extraction process, consequently improving the extraction efficiency of Ga-68. The high effectiveness of Zn(NO_3_)_2_ as a salting-out agent in solvent extraction has been described before (Qi 2018). We also observed that higher levels of radioactivity, and the corresponding increase in radiolysis of the target solution after cyclotron irradiation, did not affect the extraction. Most likely the BPHA has sufficient radiation resistance to withstand breakdown during the short contact time, enabling Ga-68 extraction with the same efficiency as at low levels of radioactivity. The membrane on the other hand, seemed to be affected by the radiation dose and the corresponding radiolysis resulting in the formation of radicals in the target solution as well as the chloroform solution. Therefore, future research about the radiation stability of the membrane is necessary in order to ensure a continuous separation of radioactive nuclides.

Martini et al. (2021) reported a maximum of 89.6% EE in batch, and 80% EE in membrane separator experiments from a 1 M Zn(NO_3_)_2_ in 0.01 M HNO_3_. They used a comparable set-up for the phase separation utilizing slug flow in a 100 cm long tubing with 0.03 inch inner diameter connected to the Zaiput membrane separator for subsequent phase separation. But the flowrates used in their study where approximately 6 times faster at 250 µL/min. An additional mixing step was also included in their set-up with simultaneous heating of the solution to 50 °C. In comparison, the results from our study show maximum EEs as high as 99.9% in batch, and 99.2% ± 0.3% in membrane separator experiments for the same zinc nitrate solution at room temperature without additional mixing, but with a slower flowrate of 40 µL/min.

Finally, the measured zinc concentration in the back-extracted solutions in our study was below 3 ppm, while Martini et al. (2021) reported 11 ppm in their study, which exceeds regulations of maximum 10 ppm, posed by the International Atomic Energy Agency TecDoc Quality Control in the Production of Radiopharmaceuticals (International Atomic Energy Agency 2018).

Our results present a robust, highly efficient strategy for the rapid extraction of Ga-68 from cyclotron-irradiated Zn(NO_3_)_2_ liquid target solutions, based on a commercially available chelator allowing for quick implementation in ^68^Ga-producing cyclotron facilities.

### Applicability in clinical setting

The developed method, presented in this study could increase the production of Ga-68 in the future. The total purification time after cyclotron irradiation can be as quick as 10 min if applied to a module of multiple microfluidic devices, since one device only has a throughput of 40 µL/min it would take significantly longer otherwise. This method can increase the amount of extracted Ga-68 by almost 25% compared to other purification methods, for the same irradiation time while potentially allowing for direct target recycling and re-irradiation. With just a single irradiation, produced activities could be tenfold higher compared to an elution of the best currently available Ge-68/Ga-68 generator. The recyclability of the target solution after the extraction could lead to more irradiations per day, potentially increasing Ga-68 production tremendously. However, before this method can be readily used in clinical settings, some issues have to be overcome. Since the radiolysis in the target solution after irradiation leads to damage in the membrane used for the separation, and to the best of our knowledge no membrane for this specific set-up exists that might have higher radiation stability, further development should focus on a different microfluidic set-up without a radiation-sensitive membrane or on the improvement of the stability of the membranes.

## Conclusion

A new two-step microfluidic extraction method for the efficient extraction of Ga-68 from liquid target solutions was developed. By using BPHA in chloroform as the extracting phase, extraction efficiencies of over 99% and back-extraction efficiencies of up to 95% could be achieved, leading to a total Ga-68 recovery of up to 94% within 5–15 min. Best results were achieved when 0.01 M HNO_3_ was used as the target solution, while only co-extracting 0.09 ± 0.06% Zn.

This method presents a very promising new approach to selectively extract Ga-68 from [^68^Zn]Zn(NO_3_)_2_ liquid targets, which decreases the time required for Ga-68 extraction, and subsequently increasing production yield, while potentially allowing for direct target recycling.

## Data Availability

The datasets generated or analyzed during the current study are included in this published article. Supplementary information is available from the corresponding author on reasonable request.

## References

[CR1] Ahmed IM, El-Nadi YA, El-Hefny NE (2013). Extraction of gallium(III) from hydrochloric acid by Cyanex 923 and Cyanex 925. Hydrometallurgy.

[CR2] Alves F, Alves VH, Neves ACB, do Carmo SJC, Nactergal B, Hellas V, et al. Cyclotron production of Ga-68 for human use from liquid targets: from theory to practice. Inn: AIP conference proceedings; 2017a.

[CR3] Alves F, Alves VHP, do Carmo SJC, Neves ACB, Silva M, Abrunhosa AJ (2017). Production of copper-64 and gallium-68 with a medical cyclotron using liquid targets. Mod Phys Lett A.

[CR4] Banerjee SR, Pomper MG (2013). Clinical applications of gallium-68. Appl Radiat Isot.

[CR5] Bhattacharya B, Mandal D, Mukherjee S (2007). Liquid–liquid extraction of gallium(III) with LIX 26. Sep Sci Technol.

[CR6] Calais J, Ceci F, Eiber M, Hope TA, Hofman MS, Rischpler C (2019). 18F-fluciclovine PET-CT and 68Ga-PSMA-11 PET-CT in patients with early biochemical recurrence after prostatectomy: a prospective, single-centre, single-arm, comparative imaging trial. Lancet Oncol.

[CR7] Council of Europe (2019). European Pharmacopoeia 10.0: gallium (68Ga) chloride solution for radiolabelling.

[CR8] Clinical Trials. https://clinicaltrials.gov. Accessed 4 Oct 2022.

[CR9] Dalmázio I, Oehlke E. Cyclohexanone microfluidic extraction of radioactive perrhenate from acid solutions. In: International nuclear Atlantic conference. Belo Horizonte, Brazil; 2017.

[CR10] Dash A, Chakravarty R (2019). Radionuclide generators: the prospect of availing PET radiotracers to meet current clinical needs and future research demands. Am J Nucl Med Mol Imaging.

[CR11] De AK, Asit KS (1967). Solvent extraction and separation of gallium(III), indium(III), and Thallium (III) with tributylphosphate. Talanta.

[CR12] Dhond PV, Khopkar SM (1976). Solvent extraction of gallium(III) with 2-thenoyltrifluoroacetone. Talanta.

[CR13] do Carmo SJC, Scott PJH, Alves F (2020). Production of radiometals in liquid targets. EJNMMI Radiopharm Chem.

[CR14] Engle JW, Lopez-Rodriguez V, Gaspar-Carcamo RE, Valdovinos HF, Valle-Gonzalez M, Trejo-Ballado F (2012). Very high specific activity 66/68Ga from zinc targets for PET. Appl Radiat Isot.

[CR15] Hoehr C, Badesso B, Morley T, Trinczek M, Buckley K, Klug J, et al. Producing radiometals in liquid targets: proof of feasibility with ^94m^Tc. Presented at the 14th international workshop on targetry and target chemistry, Playa del Carmen, Mexico; 2012. p. 56–60.

[CR16] Inoue K, Baba Y, Yoshizuka K (1988). Solvent extraction equilibria of gallium(III) with acidic organophosphorus compounds from aqueous nitrate media. Solvent Extr Ion Exch.

[CR17] International Atomic Energy Agency (2018). Quality control in the production of radiopharmaceuticals. Quality control of 68Ga produced by cyclotron.

[CR18] Isabel M, Prata M (2012). Gallium-68: a new trend in PET radiopharmacy. Curr Radiopharm.

[CR19] Katsuta S, Okai M, Yoshimoto Y, Kudo Y (2012). Extraction of gallium(III) from hydrochloric acid solutions by trioctylammonium-based mixed ionic liquids. Anal Sci.

[CR20] Kratochwil C, Flechsig P, Lindner T, Abderrahim L, Altmann A, Mier W (2019). ^68^Ga-FAPI PET/CT: tracer uptake in 28 different kinds of cancer. J Nucl Med.

[CR21] Lambrecht RM (1983). Radionuclide generators. Radiochim Acta.

[CR22] Lee M, Ahn J, Lee E (2002). Solvent extraction separation of indium and gallium from sulphate solutions using D2EHPA. Hydrometallurgy.

[CR23] Lowis C, Ferguson S, Paulßen E, Hoehr C (2021). Improved Sc-44 production in a siphon-style liquid target on a medical cyclotron. Appl Radiat Isot.

[CR24] Lyle SJ, Shendrikar AD (1965). A separation scheme for gallium, indium, thallium, germanium, tin and lead by solvent extraction with N-benzoyl-N-phenylhydroxylamine. Anal Chim Acta.

[CR25] Maecke HR, André JP. 68Ga-PET radiopharmacy: a generator-based alternative to 18F-radiopharmacy. In: Ernst Schering research foundation workshop. 2007. p. 215–42. 10.1007/978-3-540-49527-7_8 17172157

[CR26] Maljkovic D, Maljkovic D, Paulin A (1990). Extraction of gallium/III/ from hydrochloric acid by diisopropyl ether and mixture diisopropyl ether-pentanol. Solvent Extr Ion Exch.

[CR27] Martini P, Adamo A, Syna N, Boschi A, Uccelli L, Weeranoppanant N (2019). Perspectives on the use of liquid extraction for radioisotope purification. Molecules.

[CR28] Martini P, Uccelli L, Duatti A, Marvelli L, Esposito J, Boschi A (2021). Highly efficient micro-scale liquid-liquid in-flow extraction of 99mTc from molybdenum. Molecules.

[CR29] Morroni L, Secco F, Venturini M, Garcia B, Leal JM (2004). Kinetics and equilibria of the interactions of hydroxamic acids with gallium(III) and indium(III). Inorg Chem.

[CR30] Oehlke E, Hoehr C, Hou X, Hanemaayer V, Zeisler S, Adam MJ (2015). Production of Y-86 and other radiometals for research purposes using a solution target system. Nucl Med Biol.

[CR31] Pandey MK, Byrne JF, Jiang H, Packard AB, Degrado TR (2014). Cyclotron production of 68 Ga via the 68 Zn(p, n) 68 Ga reaction in aqueous solution. Am J Nucl Med Mol Imaging.

[CR32] Pandey MK, Engelbrecht HP, Byrne JP, Packard AB, DeGrado TR (2014). Production of 89Zr via the 89Y(p, n)89Zr reaction in aqueous solution: effect of solution composition on in-target chemistry. Nucl Med Biol.

[CR33] Pandey MK, Byrne JF, Schlasner KN, Schmit NR, DeGrado TR (2019). Cyclotron production of 68Ga in a liquid target: effects of solution composition and irradiation parameters. Nucl Med Biol.

[CR34] Pedersen KS, Nielsen KM, Fonslet J, Jensen M, Zhuravlev F (2019). Separation of radiogallium from zinc using membrane-based liquid-liquid extraction in flow: experimental and COSMO-RS studies. Solvent Extr Ion Exch.

[CR35] Qi D. Extractants used in solvent extraction-separation of rare earths: extraction mechanism, properties, and features. In: Hydrometallurgy of rare earths; 2018. p. 187–389.

[CR36] Radiopharma. Eckert & Ziegler extends gallium-68 generator portfolio in the United States. 2021. https://radiopharma.com/news/eckert-ziegler-extends-gallium-68-generator-portfolio-in-the-united-states/. Accessed 23 Jun 2022.

[CR37] Riedel A (1973). Systematic study of the extraction of metals using N-benzoyl-N-phenylhydroxylamine. J Radioanal Nucl Chem.

[CR38] Riga S, Cicoria G, Pancaldi D, Zagni F, Vichi S, Dassenno M (2018). Production of Ga-68 with a general electric PETtrace cyclotron by liquid target. Phys Med.

[CR39] Rodnick ME, Sollert C, Stark D, Clark M, Katsifis A, Hockley BG, Parr DC, Frigell J, Henderson BD, Abghari-Gerst M, Piert MR, Fulham MJ, Eberl S, Gagnon K, Scott PJH (2020). Cyclotron-based production of 68Ga, [68Ga]GaCl3, and [68Ga]Ga-PSMA-11 from a liquid target. EJNMMI Radiopharm Chem..

[CR40] Rösch F (2013). Past, present and future of 68Ge/68Ga generators. Appl Radiat Isot.

[CR41] Sanchez-Crespo A (2013). Comparison of gallium-68 and fluorine-18 imaging characteristics in positron emission tomography. Appl Radiat Isot.

[CR42] Siikanen J, Jussing E, Milton S, Steiger C, Ulin J, Jonsson C (2021). Cyclotron-produced 68Ga from enriched 68Zn foils. Appl Radiat Isot.

[CR43] Song SJ, Le MN, Lee MS (2020). Separation of gallium(III) and indium(III) by solvent extraction with ionic liquids from hydrochloric acid solution. Processes.

[CR44] Trapp S, Lammers T, Paulssen E, de Kruijff R. Rapid, automated radionuclide separation with high yield—towards microfluidic solvent extraction for 61,64,67Cu production. In: European research reactor conference 2023 proceedings. Antwerp; 2023. p. 177–84.

[CR45] U.S. Food and Drug Administration: FDA approves first PSMA targeted PET imaging drug for men with Prostate cancer. https://www.fda.gov/news-events/press-announcements/fda-approves-first-psma-targeted-pet-imaging-drug-men-prostate-cancer. Accessed 1 Nov 2022.

[CR46] Vadasdi GK (1969). The extraction of gallium with cupferron. Anal Chim Acta.

[CR47] Velikyan I (2015). 68Ga-based radiopharmaceuticals: production and application relationship. Molecules.

[CR48] Verweij Wi. CHEAQS Next (0.2.1.8).

[CR49] Zaiput Flow technologies. https://www.zaiput.com/product/liquid-liquid-gas-separators/. Accessed 2 Oct 2022.

[CR50] Zhuravlev F, Gulzar A, Falborg L (2022). Recovery of gallium-68 and zinc from HNO3-based solution by liquid–liquid extraction with arylamino phosphonates. Molecules.

